# Supplementary figures and images for: Spatio-temporal modelling of the first Chikungunya epidemic in an intra-urban setting: The role of socioeconomic status, environment and temperature
Source: PLoS Negl Trop Dis. 2021 Jun 18;15(6):e0009537. doi: 10.1371/journal.pntd.0009537 (PMC8244893; doi:10.1371/journal.pntd.0009537)

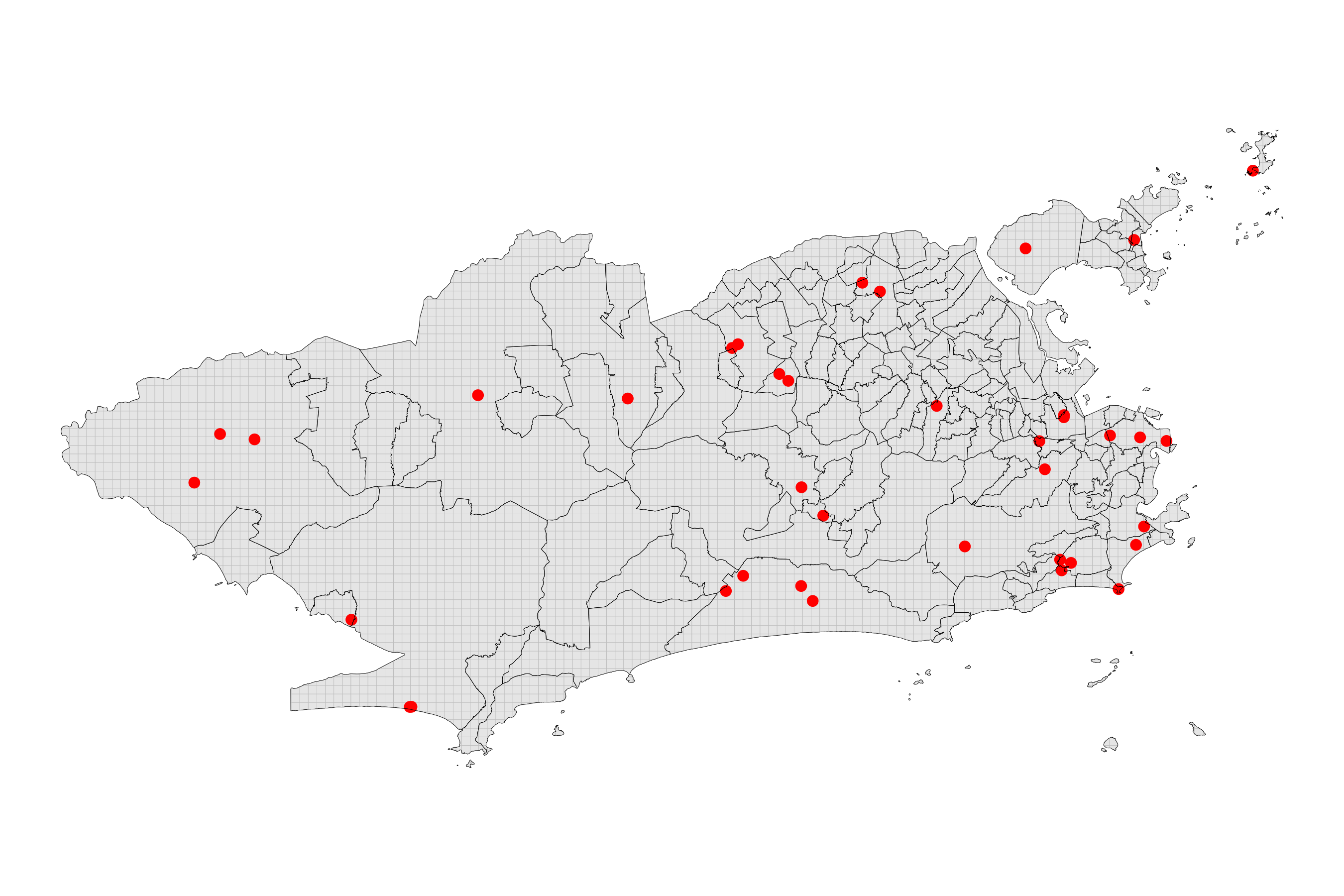

Supplement: S1 Fig — Maps created using R version 3.6.1. Map layers by Instituto Pereira Passos (https://www.data.rio/). (PNG) [file pntd.0009537.s001.png]

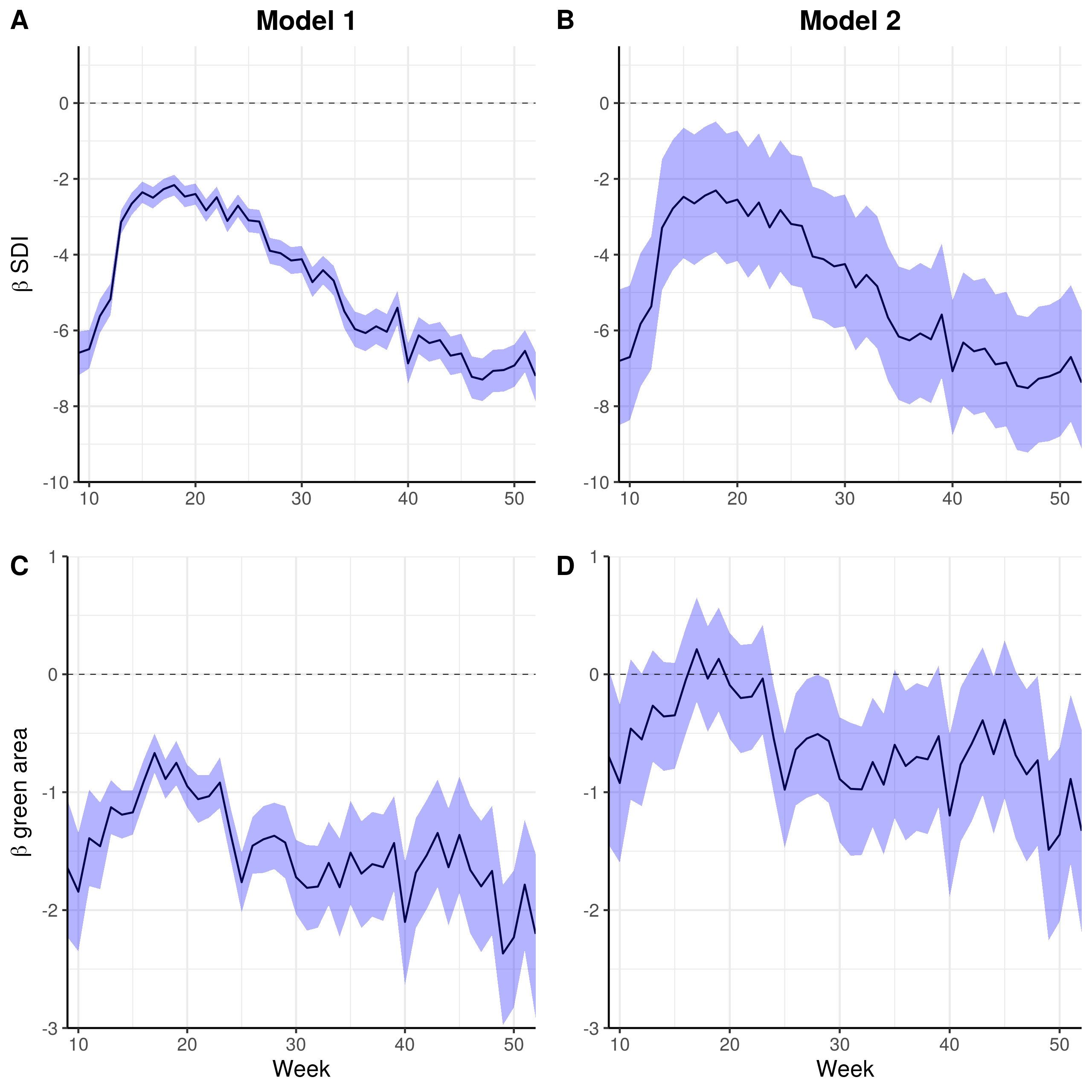

Supplement: S2 Fig — Time-varying coefficients (in the log scale, mean and 90% credible interval) for sociodevelopment index (SDI) (A,B) and proportion of green areas (C,D) without (model 1) and with (model 2) spatial dependency, for chikungunya cases from weeks 9 to 52 2016, Rio de Janeiro city, Brazil. (PNG) [file pntd.0009537.s002.png]

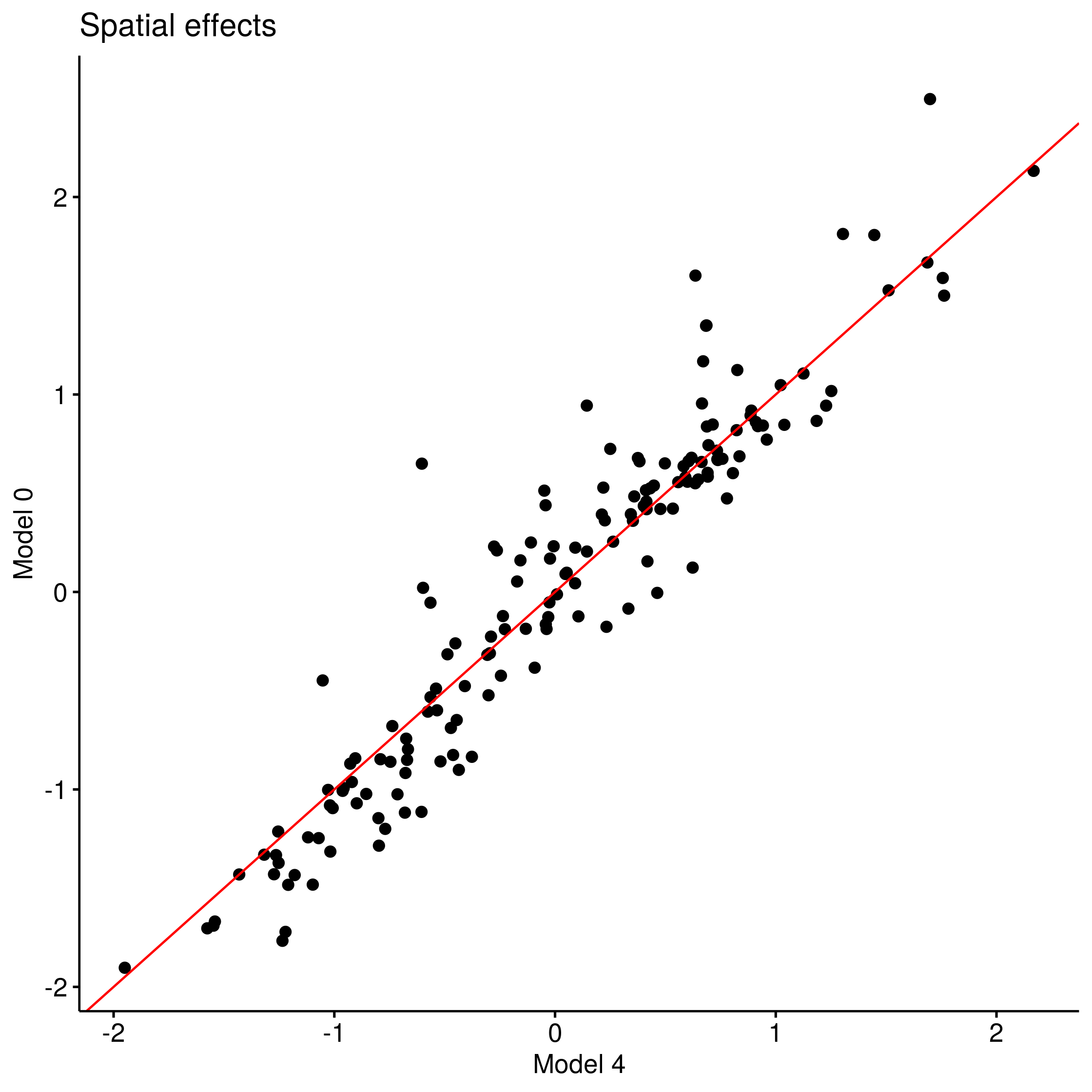

Supplement: S3 Fig — (PNG) [file pntd.0009537.s003.png]

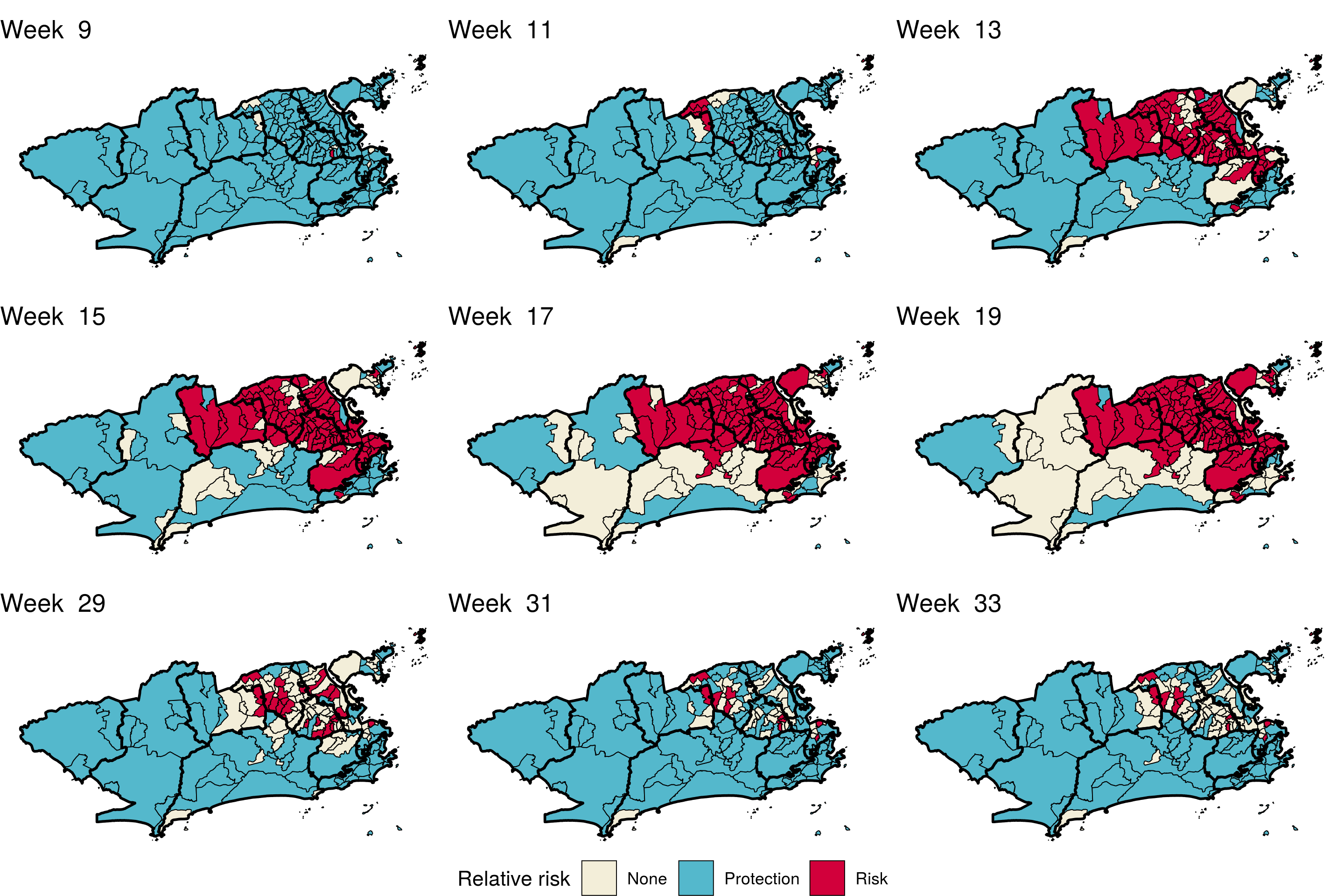

Supplement: S5 Fig — Risk: 90%CI >1. Protection: 90%CI <1. None: 90%CI includes 1. Maps created using R version 3.6.1. Map layers by Instituto Pereira Passos (https://www.data.rio/). (PNG) [file pntd.0009537.s005.png]
