# Supplementary material for: Spatio-temporal modelling of the first Chikungunya epidemic in an intra-urban setting: The role of socioeconomic status, environment and temperature
Source: PLoS Negl Trop Dis. 2021 Jun 18;15(6):e0009537. doi: 10.1371/journal.pntd.0009537 (PMC8244893; doi:10.1371/journal.pntd.0009537)
Supplement: S4 Fig — (PDF) [file pntd.0009537.s004.pdf]

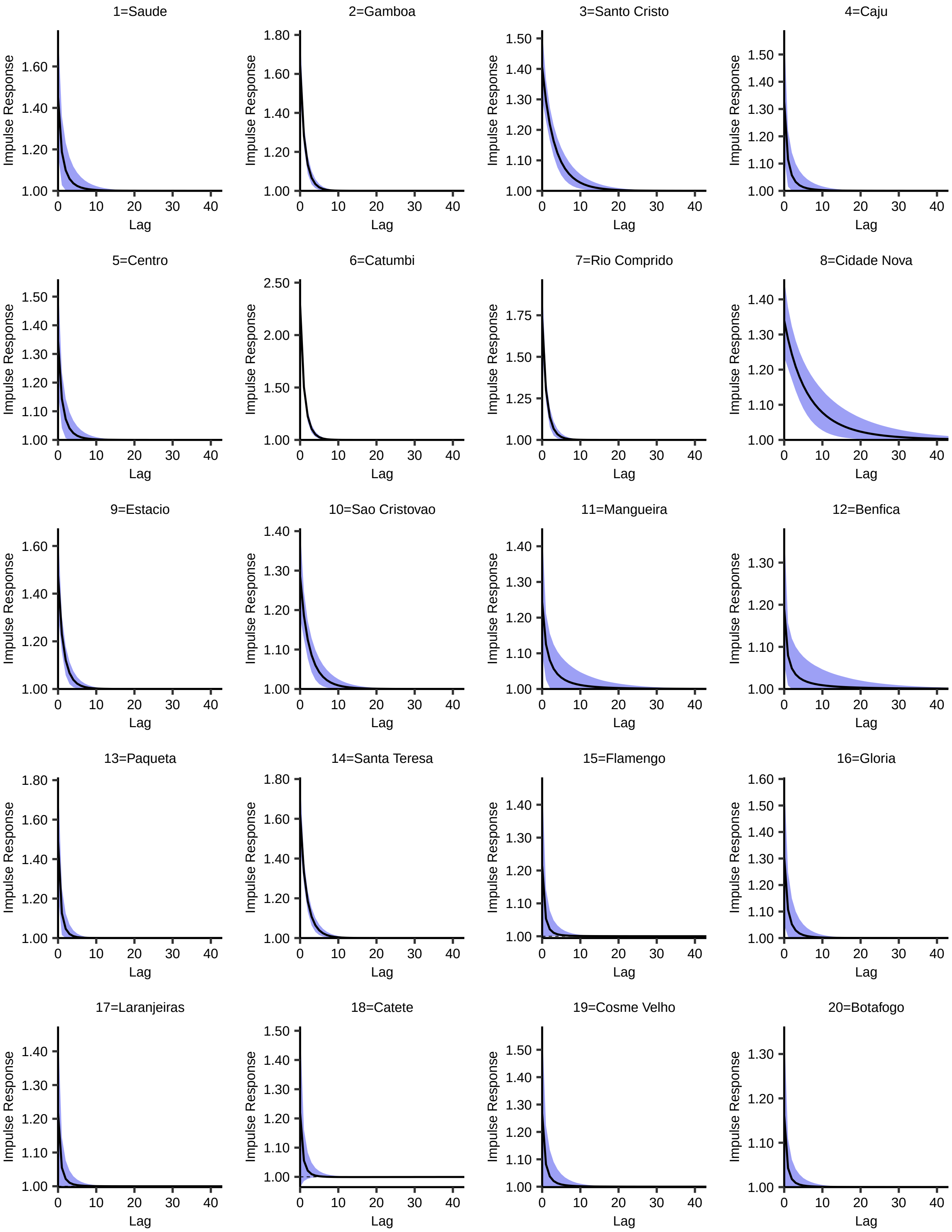
1=Saude
2=Gamboa
3=Santo Cristo
4=Caju
5=Centro
6=Catumbi
7=Rio Comprido
8=Cidade Nova
9=Estacio
10=Sao Cristovao
11=Mangueira
12=Benfica
13=Paqueta
14=Santa Teresa
15=Flamengo
16=Gloria
17=Laranjeiras
18=Catete
19=Cosme Velho
20=Botafogo
Impulse Response
Lag

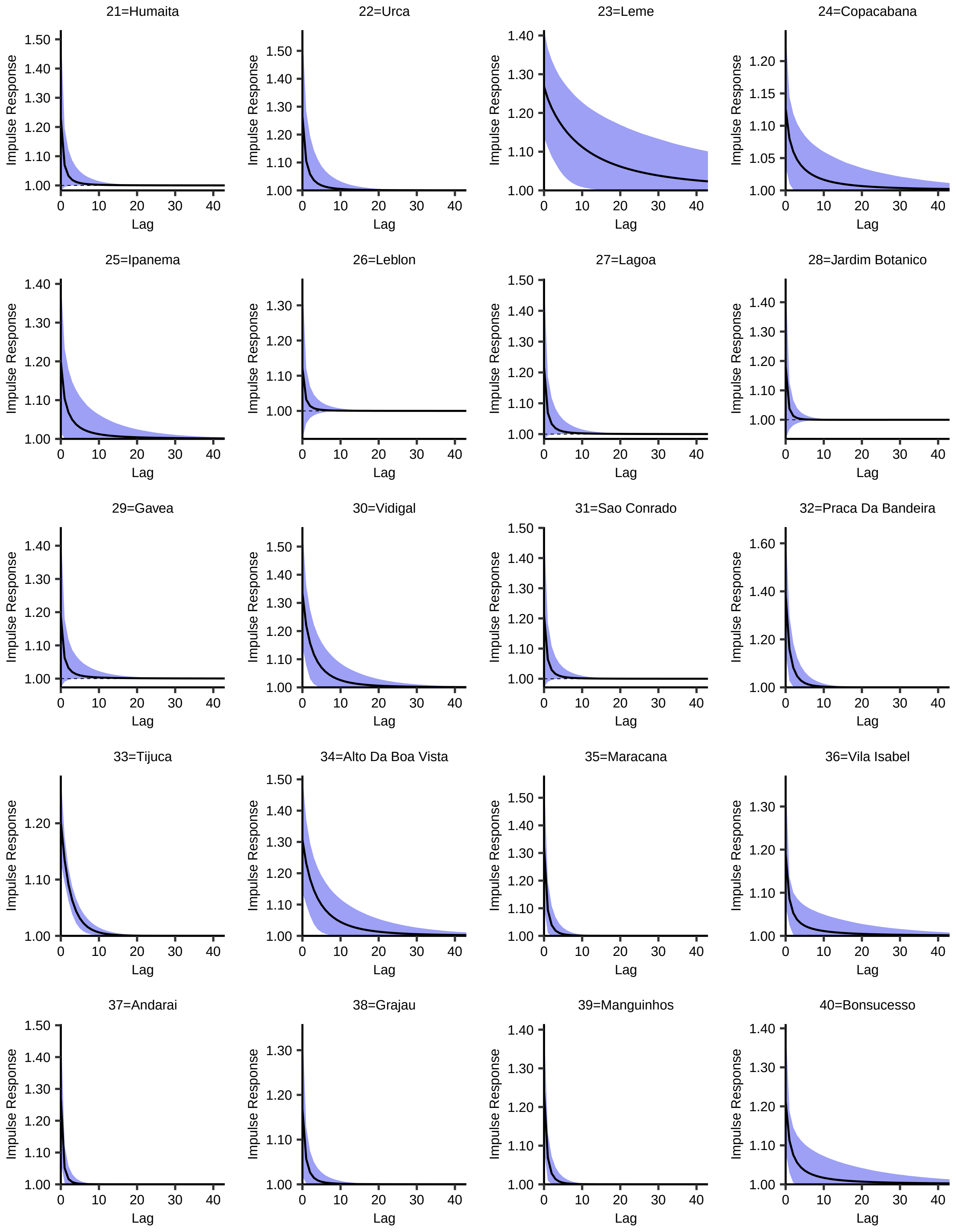
21=Humaita
22=Urca
23=Leme
24=Copacabana
25=Ipanema
26=Leblon
27=Lagoa
28=Jardim Botanico
29=Gavea
30=Vidigal
31=Sao Conrado
32=Praca Da Bandeira
33=Tijuca
34=Alto Da Boa Vista
35=Maracana
36=Vila Isabel
37=Andarai
38=Grajau
39=Manguinhos
40=Bonsucesso
Impulse Response
Lag

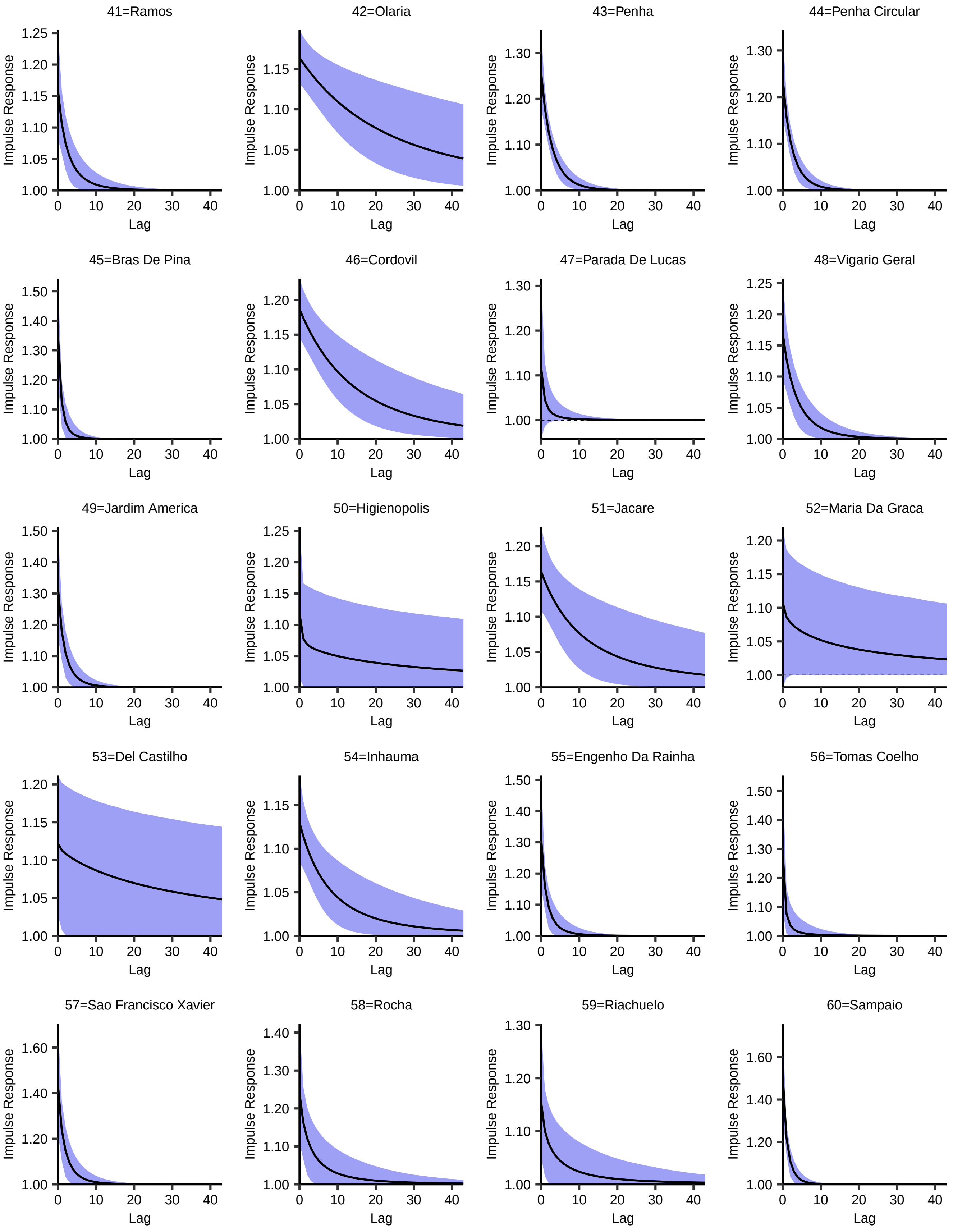
41=Ramos
42=Olaria
43=Penha
44=Penha Circular
45=Bras De Pina
46=Cordovil
47=Parada De Lucas
48=Vigario Geral
49=Jardim America
50=Higienopolis
51=Jacare
52=Maria Da Graca
53=Del Castilho
54=Inhauma
55=Engenho Da Rainha
56=Tomas Coelho
57=Sao Francisco Xavier
58=Rocha
59=Riachuelo
60=Sampaio
Impulse Response
Lag
0
10
20
30
40

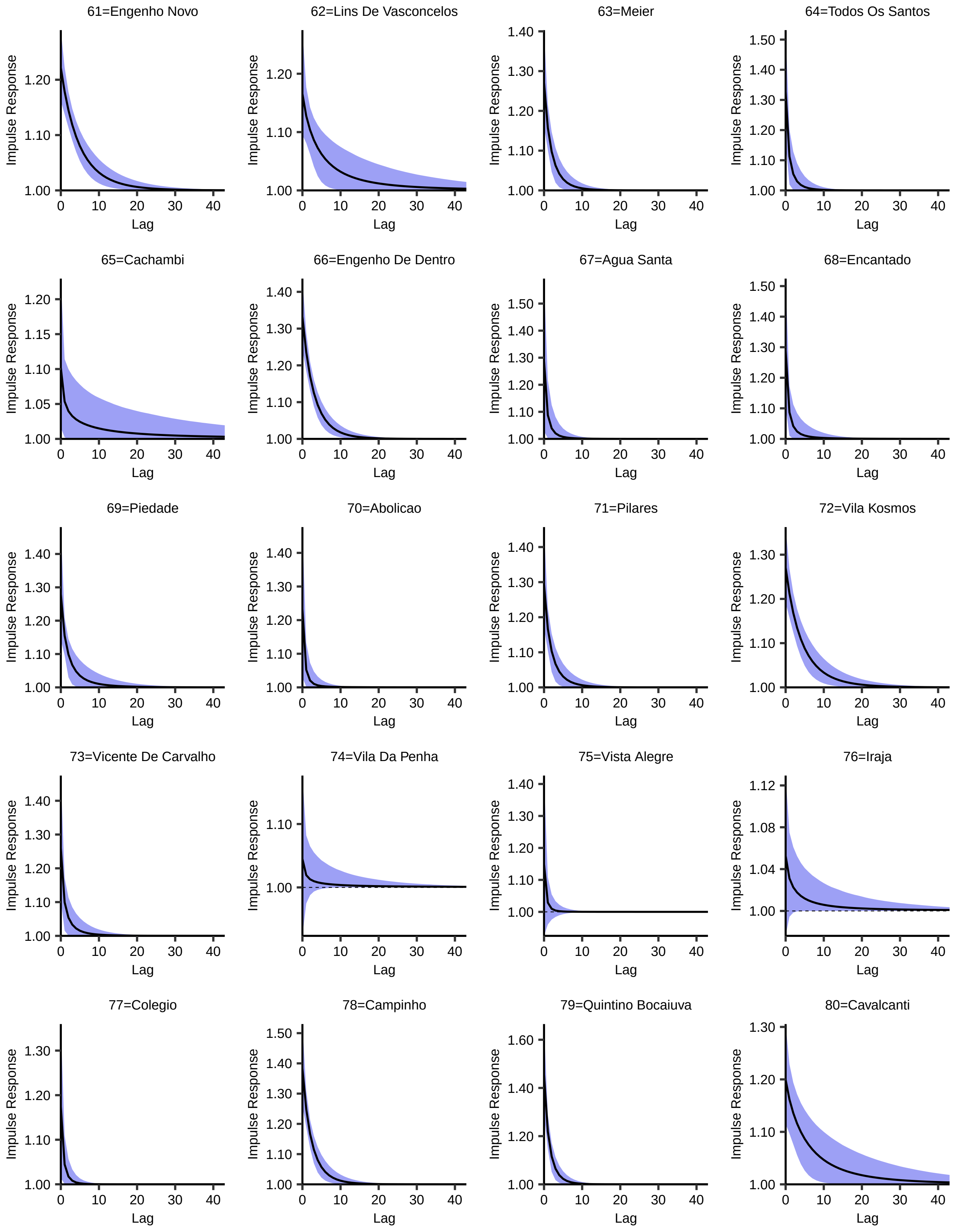
61=Engenho Novo
62=Lins De Vasconcelos
63=Meier
64=Todos Os Santos
65=Cachambi
66=Engenho De Dentro
67=Agua Santa
68=Encantado
69=Piedade
70=Abolicao
71=Pilares
72=Vila Kosmos
73=Vicente De Carvalho
74=Vila Da Penha
75=Vista Alegre
76=Iraja
77=Colegio
78=Campinho
79=Quintino Bocaiuva
80=Cavalcanti
Impulse Response
Lag

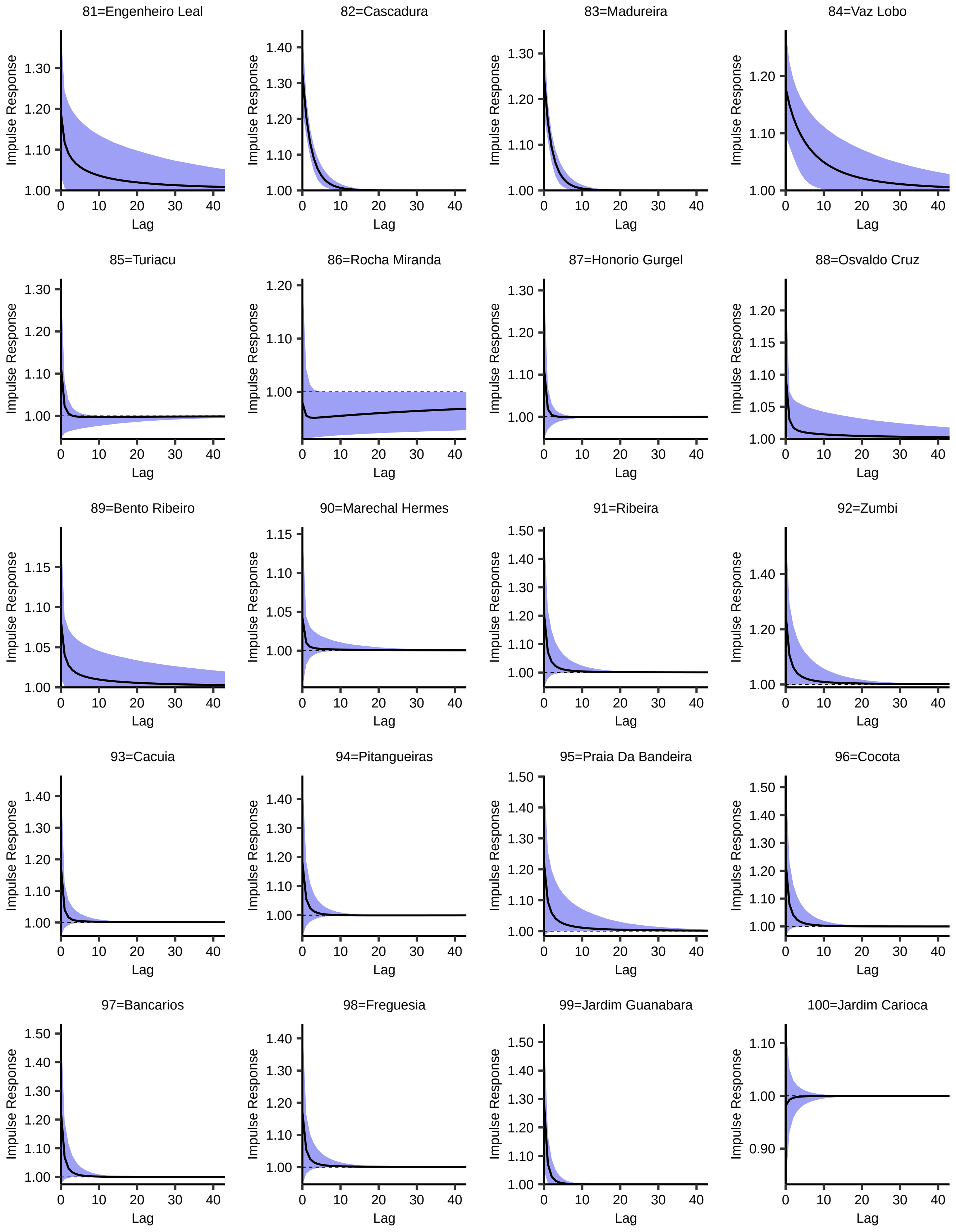
81=Engenheiro Leal
82=Cascadura
83=Madureira
84=Vaz Lobo
85=Turiacu
86=Rocha Miranda
87=Honorio Gurgel
88=Osvaldo Cruz
89=Bento Ribeiro
90=Marechal Hermes
91=Ribeira
92=Zumbi
93=Cacuia
94=Pitangueiras
95=Praia Da Bandeira
96=Cocota
97=Bancarios
98=Freguesia
99=Jardim Guanabara
100=Jardim Carioca
Impulse Response
Lag

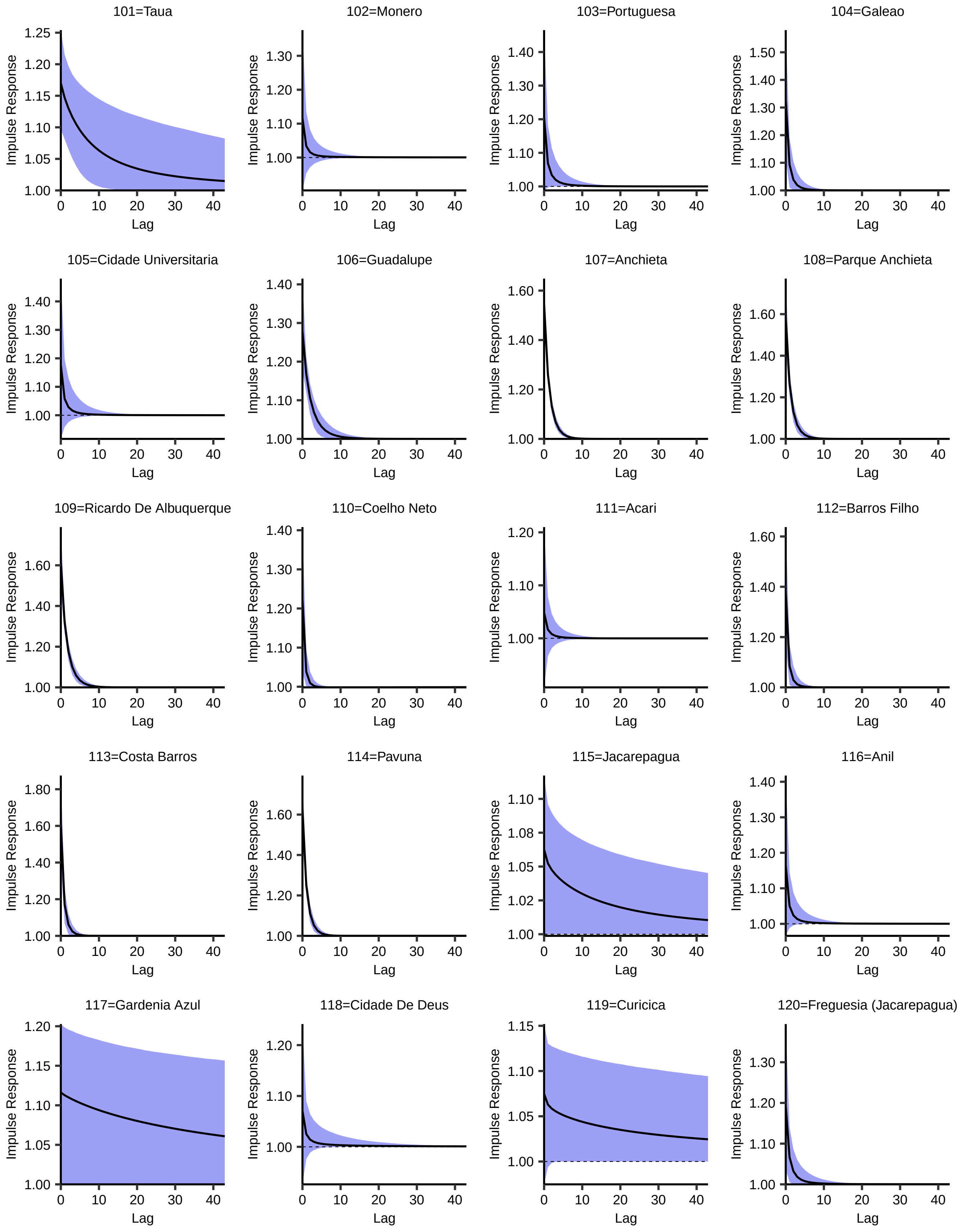

101=Taua
102=Monero
103=Portuguesa
104=Galeao
105=Cidade Universitaria
106=Guadalupe
107=Anchieta
108=Parque Anchieta
109=Ricardo De Albuquerque
110=Coelho Neto
111=Acari
112=Barros Filho
113=Costa Barros
114=Pavuna
115=Jacarepagua
116=Anil
117=Gardenia Azul
118=Cidade De Deus
119=Curicica
120=Freguesia (Jacarepagua)
Impulse Response
Lag

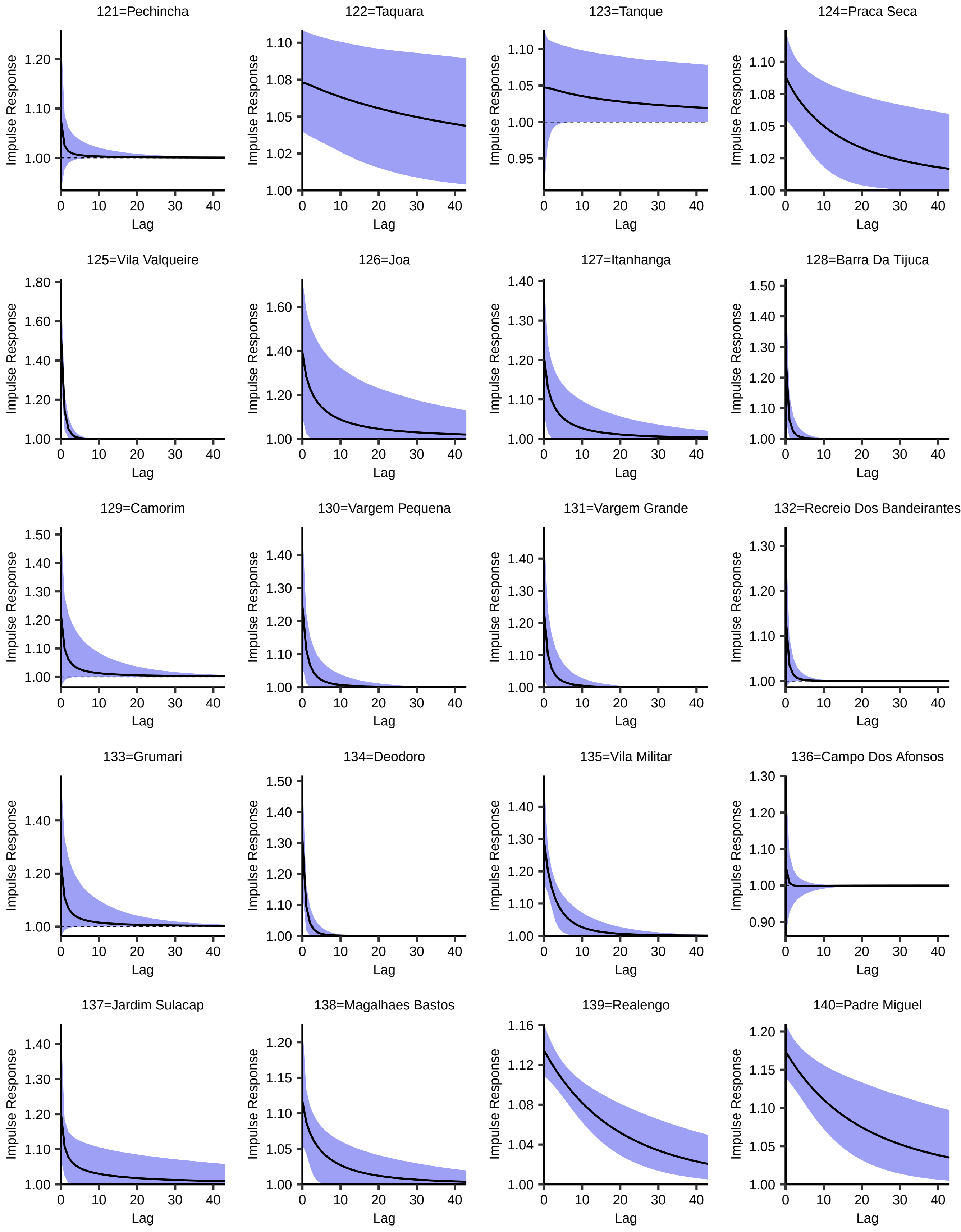
121=Pechincha
122=Taquara
123=Tanque
124=Praca Seca
125=Vila Valqueire
126=Joa
127=Itanhanga
128=Barra Da Tijuca
129=Camorim
130=Vargem Pequena
131=Vargem Grande
132=Recreio Dos Bandeirantes
133=Grumari
134=Deodoro
135=Vila Militar
136=Campo Dos Afonsos
137=Jardim Sulacap
138=Magalhaes Bastos
139=Realengo
140=Padre Miguel
Impulse Response
Lag
0 10 20 30 40
1.00 1.10 1.20
1.00 1.02 1.05 1.08 1.10
0.95 1.00 1.05 1.10
1.00 1.02 1.05 1.08 1.10
1.00 1.20 1.40 1.60 1.80
1.00 1.20 1.40 1.60
1.00 1.10 1.20 1.30 1.40
1.00 1.10 1.20 1.30 1.40 1.50
1.00 1.10 1.20 1.30 1.40 1.50
1.00 1.10 1.20 1.30 1.40
1.00 1.10 1.20 1.30 1.40
1.00 1.10 1.20 1.30
1.00 1.20 1.40
1.00 1.10 1.20 1.30 1.40 1.50
1.00 1.10 1.20 1.30 1.40
0.90 1.00 1.10 1.20 1.30
1.00 1.10 1.20 1.30 1.40
1.00 1.05 1.10 1.15 1.20
1.00 1.04 1.08 1.12 1.16
1.00 1.05 1.10 1.15 1.20

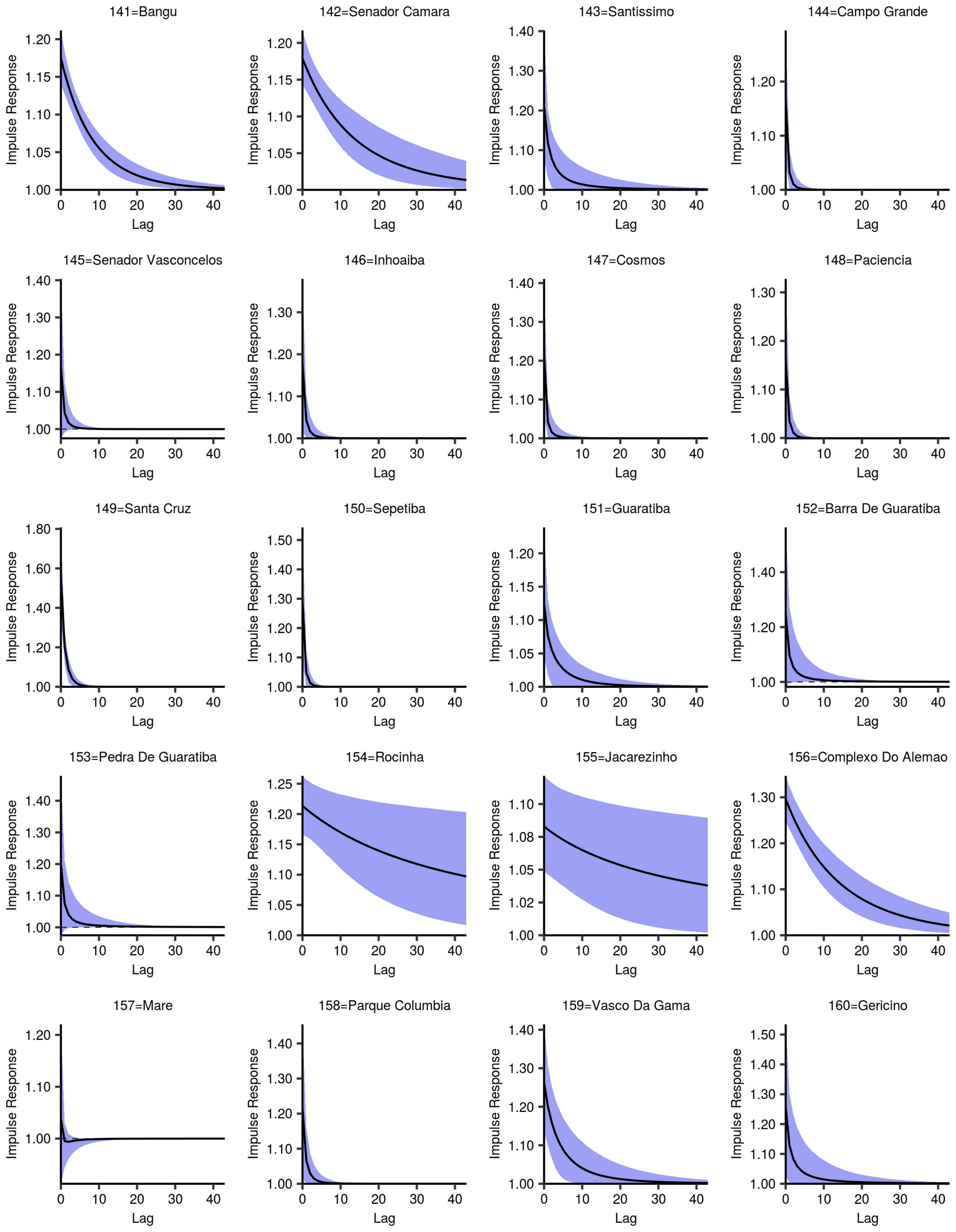
141=Bangu
142=Senador Camara
143=Santissimo
144=Campo Grande
145=Senador Vasconcelos
146=Inhoaiba
147=Cosmos
148=Paciencia
149=Santa Cruz
150=Sepetiba
151=Guaratiba
152=Barra De Guaratiba
153=Pedra De Guaratiba
154=Rocinha
155=Jacarezinho
156=Complexo Do Alemao
157=Mare
158=Parque Columbia
159=Vasco Da Gama
160=Gericino
Impulse Response
Lag
