# Supplementary material for: Spatio-temporal modelling of the first Chikungunya epidemic in an intra-urban setting: The role of socioeconomic status, environment and temperature
Source: PLoS Negl Trop Dis. 2021 Jun 18;15(6):e0009537. doi: 10.1371/journal.pntd.0009537 (PMC8244893; doi:10.1371/journal.pntd.0009537)
Supplement: S1 Appendix — (PDF) [file pntd.0009537.s008.pdf]

S1 Appendix

		WAIC
Model 0	$\log(\mu_{i,t}) = \log(e_i) + \beta_0 + \phi_i$	34114.6
Model 1	$\log(\mu_{i,t}) = \log(e_i) + \beta_0 + X'_i\beta_{k,t}$	26418.3
Model 2	$\log(\mu_{i,t}) = \log(e_i) + \beta_0 + X'_i\beta_{k,t} + \phi_i$	19656.6
Model 3	$\log(\mu_{i,t}) = \log(e_i) + \beta_0 + U_{i,t} + \phi_i$<br>$U_{i,t} = \rho_i U_{i,t-1} + \zeta_i Temperature_{i,t}$	*
Model 4	$\log(\mu_{i,t}) = \log(e_i) + \beta_0 + X'_i\beta_{k,t} + U_{i,t} + \phi_i$<br>$U_{i,t} = \rho_i U_{i,t-1} + \zeta_i Temperature_{i,t}$	17934.8

* Model 3 showed lack of convergence.
