# Supplementary material for: Spatio-temporal modelling of the first Chikungunya epidemic in an intra-urban setting: The role of socioeconomic status, environment and temperature
Source: PLoS Negl Trop Dis. 2021 Jun 18;15(6):e0009537. doi: 10.1371/journal.pntd.0009537 (PMC8244893; doi:10.1371/journal.pntd.0009537)
Supplement: S2 Appendix — (PDF) [file pntd.0009537.s009.pdf]

S2 Appendix

Model checking

As suggested by a reviewer, we first investigated model adequacy through the Bayesian mid p-value. Initially, we follow Marshall and Spiegelhalter (2003), and compare the actual observation $y_{i,t}$ with the predictive posterior distribution $p(Y_{i,t}^{rep} \mid \mathbf{y}_{-[i,t]})$, where $Y_{i,t}^{rep}$ is a replicate of the observation in neighbourhood i and week t, and $\mathbf{y}_{-[i,t]}$ denotes the set of observations excluding the one in neighbourhood i and week t. To check how extreme the observed value is under the predictive posterior distribution generated by a particular model, the mid p-value for each observation is computed as

$$p(Y_{i,t}^{rep} < y_{i,t} \mid \mathbf{y}_{-[i,t]}) + 0.5p(Y_{i,t}^{rep} = y_{i,t} \mid \mathbf{y}_{-[i,t]}). \tag{1}$$

This suggests that this comparison should be made through cross-validation; that is, we should exclude one observation at a time, fit the models, and compute the p-value each time. Because of the large sample size we have ($N \times T = 160 \times 44$ observations), and the computational burden to fit the proposed models, this approach would be extremely time consuming (Marshall and Spiegelhalter, 2003). Once a sample from the posterior distribution of the parameters of a particular model is available, Marshall and Sipegelhalter (2003) propose a mixed predictive method to approximate the posterior distribution excluding one observation at a time, $p(\Theta \mid \mathbf{y}_{-[i,t]})$, where Θ represents the parameter vector of interest.

Panels of Figure 1 show the estimated mid p-values. Clearly Model 4 provides a distribution that has high probability mass around 0.5. In particular, 13.2% (933 out

of 160×44) of the mid p-values are smaller than 0.1 or greater than 0.9, suggesting reasonable fit of the model. As pointed out by Gelman (2013), Bayesian p-values tend to have distributions more concentrated near 0.5. One of the reasons might be due to weakly informative priors, such that the center of the posterior predictive distribution will be close to $y_{i,t}$.

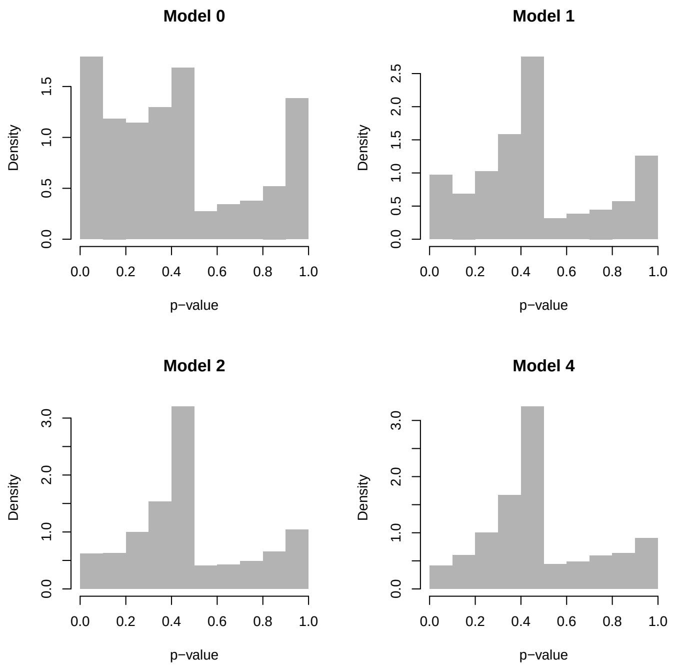

(a) Histograms

Figure 1: Distribution of the Bayesian p-values under the different fitted models.

To further investigate model adequacy we followed Czado *et al.* (2009) and computed the Probability Integral Transform (PIT). Based on a sample of size L from the posterior distribution of the parameter vector for each model, the cumulative predictive probabilities to compute the PIT were estimated as the mean of $P(Y_{i,t} < y_{obs_{i,t}} \mid \mu_{i,t}^{(l)})$, where $l = 1, \cdots, L$ and $y_{obs_{i,t}}$ denotes the observed count in neighbourhood i and week t. Deviations from the Uniform(0,1) distribution indicate poor fitting. As described in Czado *et al.* (2009) if the PIT is U-shaped this indicates an overdispersed predictive distribution. On the other hand, if the PIT is inversely U-shaped this indicates a underdispersed predictive distribution. A uniform PIT is indication of a well calibrated predictive dis-

tribution. Panels of Figure 2 show the nonrandomized PIT histograms under each of the fitted models. According to the PIT, model 4 provides the most calibrated fitted values and suggest adequate fitting.

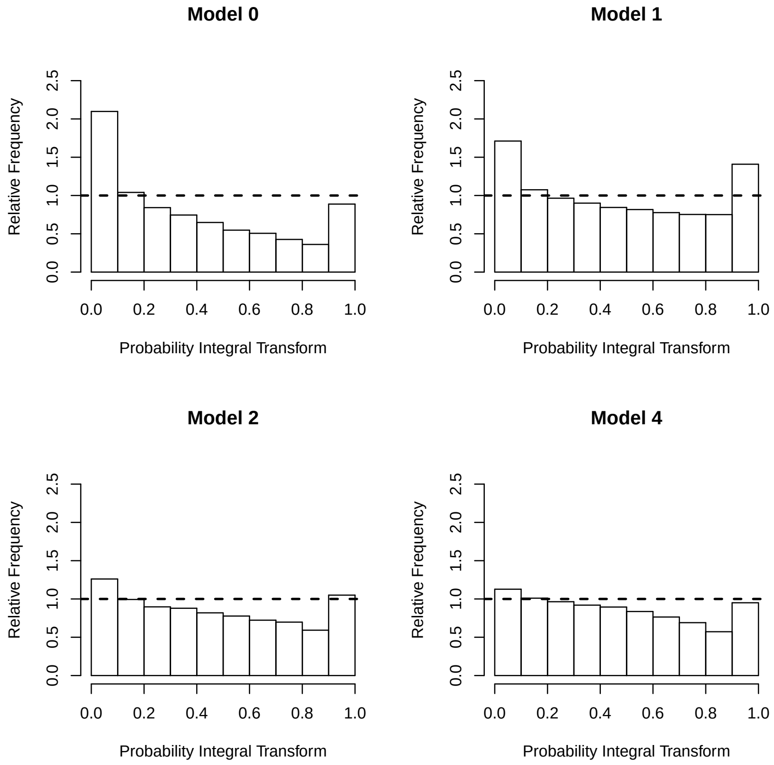

Figure 2: Nonrandomized PIT histograms computed as proposed in Czado *et al.* (2009). Deviations from the Uniform(0,1) distribution (dashed line) indicate poor fititng.

To showcase the fitted and observed values from Model 4, Panels of Figure 3 show the posterior summary of the fitted values together with the respective observed values for the same neighborhoods shown in Figure 8 of the main text. These plots were chosen because they represent well the fitted values obtained for each of the $n = 160$ neighborhoods.

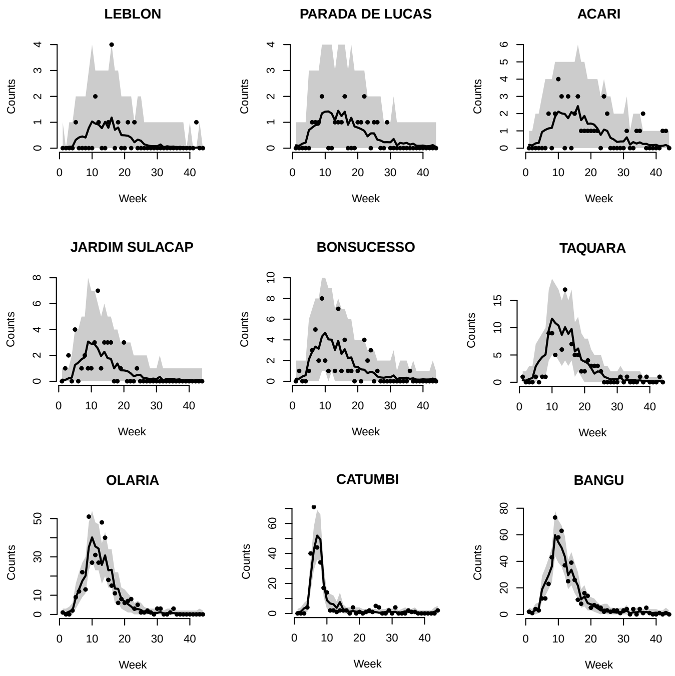

Figure 3: Posterior summaries (mean: solid line; shaded area: pointwise 95% credible interval), under Model 4, together with observed number of cases of chikungunya (solid circles) for the same nine neighbourhoods displayed in Figure 8 of the main text.

References

Czado, C., Gneiting, T. and Held, L. (2009) Predictive model assessment for count data. Biometrics, 65: 1254–1261.

Gelman, A. (2013) Two simple examples for understanding posterior p-values whose distributions are far from uniform. Electronic Journal of Statistics, 7, 2595-2602.

Marshall, E.C. and Spiegelhalter, D.J. (2003), Approximate cross–validatory predictive checks in disease mapping models. Statist. Med., 22: 1649–1660.
