# Supplementary material for: Spatio-temporal modelling of the first Chikungunya epidemic in an intra-urban setting: The role of socioeconomic status, environment and temperature
Source: PLoS Negl Trop Dis. 2021 Jun 18;15(6):e0009537. doi: 10.1371/journal.pntd.0009537 (PMC8244893; doi:10.1371/journal.pntd.0009537)
Supplement: Attachment — Submitted filename: response_to_comment_may04.pdf [file pntd.0009537.s010.pdf]

Dr. Pedro F. C. Vasconcelos
Deputy Editor
PLOS Neglected Tropical Diseases

30 April 2021

Dear Dr. Vasconcelos,

Thank you for considering for publication our manuscript entitled *"Spatio-temporal modelling of the first Chikungunya epidemic in an intra-urban setting: the role of socioeconomic status, environment and temperature"*. I am pleased to submit a new version of the manuscript after consideration of the reviewers' comments.

We would also like to thank the reviewers for their time spent on reviewing our manuscript and their comments helping us improving the manuscript.

Please find below our responses to reviewer comments including a description of the changes made in the manuscript.

Sincerely,
Laís Picinini Freitas, PhD
Fundação Oswaldo Cruz (FIOCRUZ)

Reviewer #1

The data source is introduced, but little discussion is given to the data generating mechanism. That is, how does the epidemic in the population relate to the observations. Is there evidence for asymptomatic and undetected transmission? Are there disparities in recording cases? This should be addressed.

We included an explanation in the "Data" section to explain how the data is generated (lines 143-146 in the new version):

> "In Brazil, all suspected chikungunya cases attending a health care facility must be notified to the Ministry of Health. This is done by a health care worker – usually, the physician – filling a form at the Notifiable Diseases Information System (*Sistema de Informação de Agravos de Notificação* – SINAN)."

We also modified and expanded the part of the "Discussion" section where we mention the limitations of the data (lines 421-431 in the new version):

> “Our study has some limitations. As for any study using passive surveillance data on *Aedes*-borne diseases, there is an uncertainty on the diagnosis of the reported cases as well as under-reporting. Because the data are generated from suspected cases attending health care facilities, asymptomatic and mild cases who do not seek medical assistance are not usually detected. However, SINAN has been implemented for decades, representing an important and robust data source for the study of *Aedes*-borne diseases in Brazil [66]. It should be noted that, in the same year, the city of Rio de Janeiro was also experiencing dengue and Zika epidemics [65]. Because of the association between Zika and severe congenital manifestations, the disease awareness around Zika may have improved the search for medical care and the reporting rates [67]. On the other hand, the simultaneous occurrence of three arbovirus epidemics may have impaired the differential diagnosis, as they cause similar symptoms.”

Line 221: It may not make a difference, but a quantitative convergence diagnostic like the Gelman and Rubin diagnostic is preferable to visual assessment.

We used two convergence criteria, as mentioned in lines 237-238 in the new version:

> “We visually inspected the chains and used the R-hat statistic to check convergence [47,50,51]”.

The Rhat was initially proposed by Gelman and Rubin [reference 50], and the version implemented in Stan is based on the paper by Vehtari et al, 2019 [reference 51]. The reference 51 was missing, and was included in the new version. All parameters of the model showed a Rhat smaller than 1.03 suggesting that convergence was attained. Besides looking at Rhat for each model, we ran four different chains starting from very different values. We looked at traceplots and the effective sample size, all these different criteria suggested that convergence was attained.

Statistical Analysis Generally: I'd be interested to see a posterior predictive p-value to assess the final model fit. I think the model is meaningful and useful even if the fit is questionable, but it would be good to higlight this kind of limitation.

Thank you for this suggestion. We included plots of the p-values under the four fitted models in the S2 Appendix. As pointed out by Gelman (2013), Bayesian p-values tend to be concentrated around 0.5, and this might be because of the weakly informative prior we assign to the parameters, such that the posterior predictive distribution will tend to be close to the observed value. To further investigate the adequacy of the model we also computed the nonrandomized Probability Integral Transform (PIT) as suggested in Czado *et al.* (2009). Model 4 was the one whose PIT was closest to a uniform (0,1) distribution. To showcase the fitted values obtained under Model 4, Figure 3 in S2 Appendix, shows the summary of the posterior predictive distribution for nine neighbourhoods together with the respective observed values. Clearly, the proposed model provides an overall adequate fit, less so for areas with

low counts. However, the contribution of those neighbourhoods to the model estimation is very limited in a scenario of over 13 thousand total cases.

We included the following in the text (lines 239-241 in the new version):

> “We also computed the Bayesian p-values of the different models and the nonrandomized Probability Integral Transform (PIT) [52] to investigate model adequacy (S2 Appendix).”

The proposed model is not dynamic in the same way that compartmental techniques are, so this is one area where limitations of the proposed method may arise.

We believe that this comment originated by the fact that we used the word “dynamic”. In our paper, we use “dynamic” in the epidemiological context of time and space variation of the incidence rate and its relationship with covariates, which is dynamic. It was not our objective to study the transmission dynamics, usually approached using compartmental techniques, but the dynamics of the disease incidence. Therefore, we believe, they are different objectives not characterizing a limitation, but a choice of method.

I applaud the authors for making their code available.

Thank you.

I found the results to be well articulated.

Thank you.

Overall the conclusions are well stated and the limitations well articulated. One area where this should be expanded is the same as mentioned previously - the difference between the techniques proposed and mechanistic models which account for transmission dynamics. To be clear, I think the authors' approach is reasonable, but it should be placed in the context of the wider stochastic epidemic modelling literature.

We appreciate the comments. The response to this comment is included above, when the comparison with mechanistic models issue was first raised.

Overall, the manuscript is well written and clear, both in terms of methodology and problem description. There are a few minor language issues to double check, mainly consisting of issues of word choice and numerical agreement. Here are a few examples - overall the writing is quite good.

Abstract
"Green" areas should be clarified. Environments with vegetation are likely heterogeneous with respect to the factors identified.

Summary
- Line 42: "transmitted viruses"
- Line 45: "status plays"
- Line 47: "improving ... is"

Introduction
- Line 61: facilitating the establishment of a
- Line 63: citation needed - antibody tests can certainly be cross-reactive between these viruses
- Line 66: "conditioned by" -> "favorable to"
- Line 73: "data regarding the"
- Line 93: "favor contact" (remove "the"), "the human" -> "humans"
- Line 96: "disease epidemics"
- Line 98: "including intrinsic conditional"

Thank you for identifying these language issues. We made a careful review of the text and made some corrections.

Regarding Line 63, we changed the text to clarify that previous exposure to DENV does not provide immunological protection against CHIKV infection (lines 67-69 in the new version):

> “Because CHIKV and DENV belong to different families, previous immunity to DENV does not protect against CHIKV infection, and the population of Rio de Janeiro can be considered equally naïve to CHIKV before 2016.”

Overall this is a strong manuscript in need of a few additional areas of discussion, and ideally some investigation of model fit (beyond the relative measures of information criteria).

Thank you very much for your careful review and comments.

Reviewer #2

> The authors present a study of the first chikungunya outbreak in Rio de Janeiro in 2016, and analyze this dataset of notified cases by neighborhood and week using Bayesian (intrinsic conditional autoregressive [ICAR]) spatio-temporal models to understand the drivers of this outbreak. They investigate effects of temperature, and include this as both a direct and a decaying effect, socioeconomic status, and green space. Covariate effects are allowed to vary over time, so that they can tease apart whether the importance of different factors changes as the epidemic progresses. I think this is a strong and helpful piece of work. The insights on how socioeconomic status of neighborhoods is tied to transmission early on in the pandemic, and as it fades, but is essentially absent during the peak, is particularly interesting. I offer the following comments in the hope they can improve the paper further:

Thank you very much for your careful review and comments.

> Socioeconomic status was based on an index, which was an average of eight normalized indicators. I am always somewhat uncomfortable with indicator variables, because the interpretation of an arithmetic average of normalized values becomes difficult to interpret – why not use a subset of the indicators directly as variables? Would it not be much more helpful to know whether it is water supply that leads to vulnerability, or sewage or garbage collection, and how important these different factors are relative to each other?

We agree with the reviewer that including separate components of the social index would be more helpful to identify where to intervene. However, these variables are usually highly correlated and, from a statistical point of view, it might be challenging to estimate the coefficients of these components with the relative risk. The advantage of using the index is that accounts for this possible correlation and provides a summary of the social conditions across the city. Moreover, our goal was to identify socioeconomic disparities and for that, in the context of the city of Rio de Janeiro, the index is more advantageous.

> Green areas: can you explain in more detail why you combined these various "natural" areas into green space for your analysis? Areas like agricultural land are not associated typically with *Ae. aegypti*, while something like canopy cover could certainly lead to more favorable microclimates for this species. (in other words, the composite variable might end up not having an effect because it combines both positive and negative land use elements).

As stated in the introduction (lines 94-96 in the new version):

> "Regarding the environment, the *Ae. aegypti* mosquitoes are highly adapted to urban settings, and the level of urbanisation is inversely correlated with the proportion of green areas [11]".

We also included the following (lines 96-97 in the new version):

> "In Rio de Janeiro city, previous studies found the *Ae. aegypti* mosquito to be more abundant in urban locations compared to rural and forested areas [12,13]."

Therefore, our goal with the "green areas" variable was to account for non-urban areas. Also, in Rio de Janeiro city, the vast majority of green areas are in the Atlantic forest in the mountain massifs. These are large areas where people do not live. As explained in lines 371-378 (new version), we believe that the lack of effect of green areas was possibly caused by spatial confounding.

> "When the spatial dependency was not included in the model, the proportion of green areas was negatively associated with the number of chikungunya cases (Fig C in S2 Fig). Such association was observed for dengue in São Paulo, where low vegetation cover areas presented higher dengue incidence rates [23]. However, with the inclusion of the spatial component, the effect of the proportion of green areas moved towards the null. This is possible due to spatial confounding, which happens when covariates that are spatially smooth are collinear with spatial random effects [60]. In Rio de Janeiro, the majority of the green areas are in the mountain massifs (Fig 2), which trespasses different neighbouring borders."

Although the minimum temperature can certainly be important, so can the maximum (e.g., temperatures that are overly hot can impair mosquitoes as well). Why only pick the minimum here?

We explained the following (lines 188-190 in the new version):

> "We decided to use the minimum temperature as in tropical climates it acts as a limiting factor for the *Ae. aegypti* activity and population [40,41]."

In addition, during early stages of the modeling, we explored models with the maximum temperature. However, these models presented worse fitting compared to the ones with minimum temperature. Considering the high correlation between both variables, we decided to include only the minimum temperature in the models.

I appreciate the mention of limitations in the discussion. For the third of those (people potentially getting infected in neighborhoods other than where they live), I'd like to see some more discussion, as with the other limitations: what do we know about this, how likely is it, how would you change your modelling approach to account for it if you think it likely is important?

We included the following in the discussion (lines 437-440 in the new version):

“This is an unavoidable limitation when using surveillance data. It is not a trivial task to identify where the person was infected when dealing with *Aedes*-borne diseases. Entomological surveillance research and human population mobility data could be explored in future studies and potentially bring insight on where the most common places of transmission are.”
